# *Clostridium difficile*: epidemiology, diagnostic and therapeutic possibilities—a systematic review

**DOI:** 10.1007/s10151-013-1081-0

**Published:** 2013-11-01

**Authors:** M. Kazanowski, S. Smolarek, F. Kinnarney, Z. Grzebieniak

**Affiliations:** 1 Second Department of General and Oncological Surgery, Wroclaw Medical University, Borowska Street 213, 50-556 Wrocław, Poland; 2 Department of General Surgery, St Michaels Hospital, Dublin, Ireland

**Keywords:** *Clostridium difficile*, Pseudomembranous colitis, Fulminant colitis, Nosocomial infections

## Abstract

This literature review looks at the epidemiology, clinical manifestations, diagnostics and current medical and surgical management of *Clostridium difficile (C. difficile)* infection. A literature search of PubMed and Cochrane database regarding *C. difficile* infection was performed. Information was extracted from 43 published articles from 2000 to the present day which met inclusion criteria. *C. difficile* is a gram-positive, anaerobic bacillus, which is widely found in the environment, especially in the soil. The occurrence of more resistant strains, which is mainly connected with the wide use of antibiotics, resulted in the rapid spread of the bacteria to different hospital departments. Particularly, elderly patients in surgical wards and intensive care units are at significant risk of developing *C. difficile* infection, which greatly increases morbidity and mortality. Symptoms of infection with *C. difficile* vary greatly. At one end of the spectrum, there are asymptomatic carriers, at the other patients with life-threatening toxic megacolon. Metronidazole is considered to be the drug of choice, but recent guidelines recommend Vancomycin. Fulminant colitis and toxic megacolon warrant surgical intervention. The optimal time for surgery is within 48 h of initiating conservative treatment without seeing a response, the development of multiple organ failure or a bowel perforation. A factor that has become increasingly important and relevant is the escalating expense of treatment for patients with *C. difficile* infection. It is, therefore, highly recommended to consider reviewing all hospital antibiotic policies and clinical guidelines that may contribute to the prevention of the infection.

## Introduction

The name “*Clostridium difficile*” (*C. difficile*) comes from the Greek word “Kloster” which means spindle. It was first mentioned in the literature in 1935 by Hall and O’Toole [1]. At first, the bacterium was given the name “Bacillus difficilis.” (Latin: difficilis, meaning difficult). This was because of the difficulty encountered isolating the bacteria and also the fact that it had a very slow growth phase during culturing. The name was subsequently changed in the 1970s to *C. difficile*.

*Clostridium difficile* is a gram-positive, anaerobic bacillus, which is found widely in the environment, especially in the soil. Despite the fact that even in the first known description of *C. difficile* the authors had mentioned its deadly effects on mice, the complete virulence of the bacterium was not properly recognized until much later. During World War II, Hambre et al. [2] observed using animal models that mice treated for gas gangrene with penicillin suffered from a very severe form of typhlitis. This in fact turned out to be even more deadly than the gangrene itself caused by *Clostridium perfringens*. This discovery led to new tests in which researchers gave rodents different kinds of antibiotics watching for the development of very similar symptoms. Green [3] used guinea pigs in his experiments, during which he was able to induce death by giving them large doses of penicillin. He then studied the stool of the dead guinea pigs and discovered the presence of cytoplasmic changes within. This was the first description of *C. difficile* toxin. It was Cohen and colleagues [4] who actually documented the connection between pseudomembranous colitis and antibiotic therapy. One year after the publication of this association, Tadesco et al. [5] had noticed that patients treated with clindamycin (almost 21 %) suffered from diarrhea and (10 %) were diagnosed with pseudomembranous colitis. This trial involved over 200 patients and was the first trial in which endoscopy was used so routinely on such a large group of patients. It led to the identification of *C. difficile* as a causative factor for multiple ailments involving the digestive system.

*Clostridium difficile* is found in 66 % of the digestive tracts of asymptomatic infants and young children. This could be secondary to the fact that not all of the receptors in the intestinal epithelium have matured completely. In adults, colonization affects about 3 % of the population. This number increases considerably during long hospital stays and postoperatively. The bacteria are present mainly in a vegetative form and are very sensitive to atmospheric oxygen. Under the influence of considerable stress, they may take the form of a spore and are thus able to survive harsh environments, such as the acid content of the stomach. With this resilience, *C. difficile* can find itself intact in the small intestine and transform itself back into a vegetative form. It can then colonize the epithelial lining of the mucosa in the digestive tract, and the problems caused by the presence of bacteria are due to several different toxins it produces. The best known are toxin A (enterotoxin) and B (cytotoxin), which under favorable conditions are produced in copious amounts. Inside the cell membrane, these toxins inactivate the transformation pathway mediated by Rho family proteins, which are responsible for the proper construction of actin cytoskeleton and the signal transduction by GTP. This affects the cell and leads to cessation from its regular cycle and apoptosis [6]. Both toxins also affect the strength of the intercellular bonds [7]. The relationship between the amount of toxins in the feces and the severity of symptoms has been demonstrated. Significant increases in toxins in the fecal load are associated with the significant deterioration of the general condition of the patient [8]. Toxin A leads to an increased secretion of fluid within the digestive tract, mucosal inflammation and structural damage. Toxin B is in most cases responsible for the major problems associated with infection. It is estimated that it has approximately 10 times more impact on the gastrointestinal mucosa than toxin A [7]. Brito et al. [7] came to the conclusion that the strains, which do not produce toxin A, are just as dangerous as those which have both toxins. There is also a hypervirulent strain in existence, which was first observed at the beginning of the twenty-first century. This particular strain is responsible for the outbreaks of highly virulent pathogens and is referred to as NAP1/BI/027. The complexity of the name is due to the different methods applied in detecting the presence of the bacteria: pulsed-field gel electrophoresis (NAP1), restriction endonuclease analysis (BI) and polymerase chain reaction (027). Type C toxin is produced by this particular pathogen. The increasing frequency, with which we now see treatment-resistant and more virulent strains of the bacteria, led the authors of this paper to review the literature on *C. difficile* infection and the treatment options available.

## Materials and methods

A literature was carried out using the electronic databases of PUBMED and Cochrane (up to December 2012) for relevant papers using the following terms: “*Clostridium difficile,*” “*Clostridium difficile* infection,” “*Clostridium difficile* treatment,” “*Clostridium difficile* colitis” and “*Clostridium difficile* fecal transplant,” limiting our search to only English language articles. The reference lists of used papers were also check and reviewed to identify publications on the same topic.

## Results

Information and data used for this publication were obtained from 43 articles which met the searching criteria.

We excluded search results for which only an abstract was available and case studies; however, we checked the references from those we found to be interesting. We included studies (review papers, meta-analysis and guidelines) which described the epidemiology and first publication about *C. difficile* infections. We compare different papers and results according to the stage and conservative treatment which was used. Data were collected from original papers referring to the possible surgical approaches, those routinely and unusually used.

Data extraction regarding epidemiology, pathophysiology, clinical manifestation with diagnosis and treatment of the infection was completed by the first author and co-reviewed by the second author.

### Epidemiology

The occurrence of diarrhea and pseudomembranous colitis significantly increased, immediately following the introduction of widely available antibiotic treatments. At first, the use of clindamycin was connected with those ailments and following this, the broad use of Penicillin. It took many more years to identify *C. difficile* as being the causative bacterium responsible for most of the symptoms associated with the wide use of antibiotics. The simple way in which the bacteria spread resulted in a significant increase in the number of infections, especially among hospitalized patients. The occurrence of more and more resistant strains resulted in the rapid spread of the bacteria to different departments, particularly the surgical wards and intensive care units but also many other medical wards. In a retrospective Canadian study, diarrhea and pseudomembranous colitis were identified as occurring 4 times more frequently in the general population in 2003 than in 1991 and 10 times more frequently than in 1938 [9]. The same study noted a significant increase in the symptoms of infection among hospitalized patients from 3 to 12 per 1,000 patients (the difference between 1991 and 2003) and up to 43 per 1,000 patients in 2004. Not only has the number of cases grown significantly over the years, but the severity has increased and the general condition of the patient has deteriorated. More and more patients, in addition to standard conservative treatment, require surgery. In another study, 10 % of patients were hospitalized in intensive care units and 2.5 % required an emergency colectomy. The mortality in this study reached 16 % [10]. Similar statistics were observed in Europe and the USA, where in 1999–2007, *C. difficile* was the main contributor to death in patients with inflammatory bowel disease [11].

Nosocomial infections are characterized by a much more severe form of the disease than in the general population, and a higher incidence of *C. difficile* infection has been noted, especially since 2000. This problem becomes particularly apparent among the patients aged over 65 years. According to different authors, the frequency of the carrier stage in patients with long hospital stays or those treated in the intensive care units ranges from 20 to 50 %, while in healthy adults, this quantity reaches only about 3 % [12]. Infected patients are often asymptomatic and although they may not feel any discomfort, they act as a reservoir for the bacteria and facilitate the spread of the pathogen among other patients. Infection spreads easily by the fecal–oral route and by direct contact with the patient (fomites), in particular through the hands of hospital personnel, clothes and stethoscopes. Patients, who during their hospital stay were already carriers, usually experience a much milder form of infection or remain asymptomatic [13]. The major risk factor for *C. difficile* infection is widespread use of antibiotics, often without the appropriate indications (Table 1). The use of antibiotics disrupts the natural colonic flora, thereby providing *C. difficile* with the opportunity to multiply and produce its toxins. Other risk factors include long duration of admission, advanced age, severe comorbidities, the use of proton pump inhibitors, enteral feeding, gastrointestinal surgery, chemotherapy and the use of tumor suppressor agents in postoperative transplant patients [14].

**Table 1:** Antibiotic groups that may predispose to *C. difficile* infection

Commonly	Occasionally	Seldom
Fluoroquinolones	Macrolides	Aminoglycosides
Clindamycin	Trimethoprim	Tetracyclines
Penicillins	Sulfonamides	Chloramphenicol
Cephalosporins		Metronidazole
		Vancomycin

### Clinical presentations

Symptoms of infection with *C. difficile* are very diverse. At one end of the spectrum, there are asymptomatic carriers at the other patients with life-threatening toxic megacolon. Infection and colonization itself are not the only prerequisite for the development of severe symptoms. What is necessary is a major disturbance of the internal bacterial flora, the risk factors which have already been mentioned and the use of antibiotics, which all have an important role in altering the intestinal flora.

#### Carrier stage

This spreads very quickly in the hospital environment, in particular among patients treated in the intensive care units and surgery departments. Most asymptomatic cases progress without any clinical manifestations. McFarland et al. [15] studied 428 patients admitted to hospital within an 11-month period. Twenty-one percent of patients, who were negative for *C. difficile* infection prior to hospitalization, were then subsequently indentified as asymptomatic carriers. Sixty-three percent remained carriers of the infection until the end of the study. According to Lawrence [16], as many as 50 % of hospitalized patients who have had no prior contact with the pathogen become carriers following lengthy hospital stays. Disruption of normal intestinal flora can easily occur with the use of antibiotics and the proliferation of bacteria within the gastrointestinal tract, leaving the intestine susceptible to the adverse influence of bacterial toxins. As previously mentioned, in the carrier stage of *C. difficile,* the severity and frequency of symptoms of the disease are limited. Research regarding the treatment of carriers currently is scarce and is not considered to be of benefit. Treatment of asymptomatic carriers is not recommended [16].

#### *C. difficile*-associated diarrhea (CDAD)

This bacterium is responsible for the majority of the cases of diarrhea in hospitals. Its presence is significantly higher in the hospital environment, where therapies that are used require a different group of antibiotics, such as clindamycin, cephalosporins, fluoroquinolones and penicillins [17]. Diarrhea usually occurs after 48–72 h after infection and is often accompanied by severe abdominal pain and cramps. There can be 10–15 bowel movements a day. A significant number of stools per day can lead to changes in the electrolyte and water balance. In patients with severe conditions, especially after surgery, CDAD increases mortality and morbidity rates.

#### *C. difficile*-associated colitis (CDAC)

**Fig. 1:**
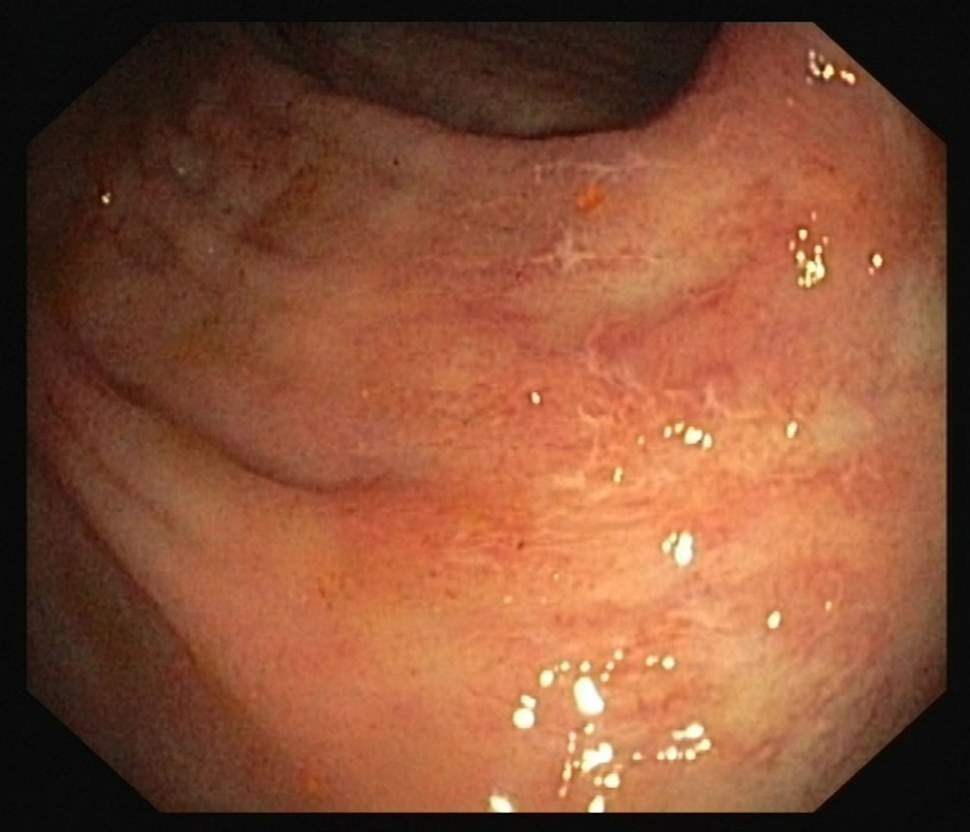
*C. difficile* colitis—picture 1 minor changes

**Fig. 2:**
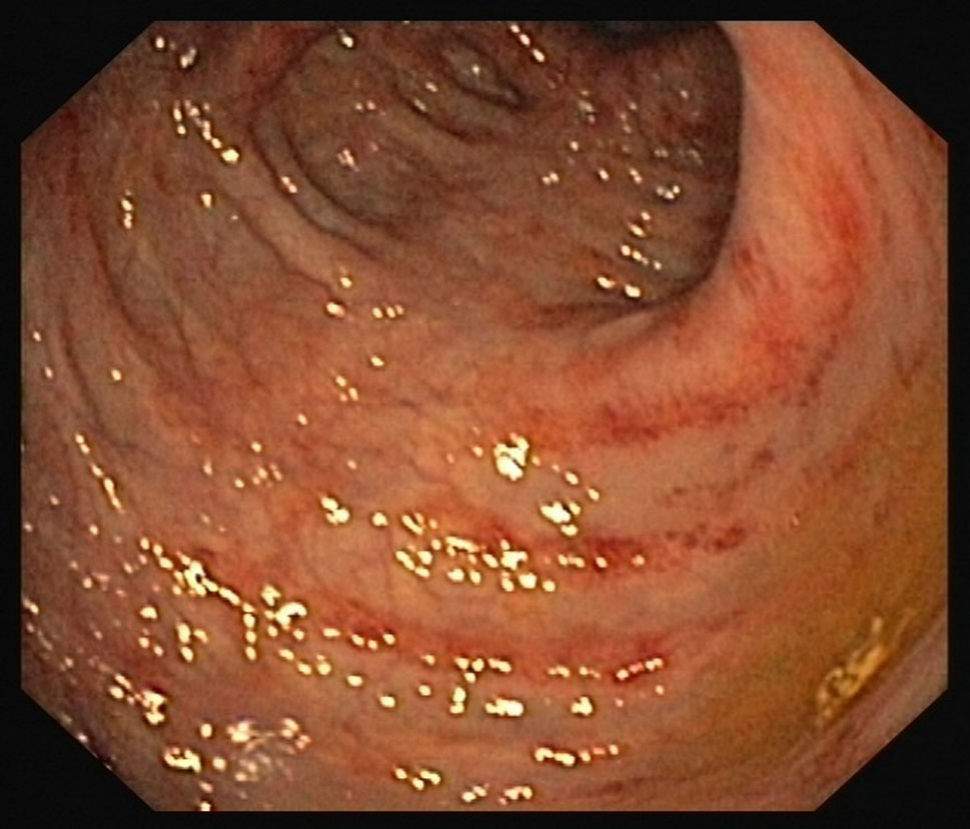
*C. difficile* colitis—picture 2 minor changes

Symptoms are very similar to those found in CDAD but also include pyrexia and leukocytosis (with an average white blood cell count of 15 × 10^9^/L). Regarding the physical examination, the most commonly elicited sign in CDAC is abdominal guarding. Other findings can include significant levels of dehydration and a positive fecal occult blood test. A colonoscopy can be helpful at an early stage as specific distinctive changes will be visible in the wall of the bowel. These include characteristic erythematous mucosa with noted friability and bleeding on contact (Figs. 1, 2). Research from Wanahita et al. documents that out of 60 patients with unexplained leukocytosis, 58 % had stool cultures positive for the bacterial toxins. In such cases, symptoms of diarrhea were observed approximately 24–48 h later [18]. Therefore, in cases of patients receiving antibiotics who have a high white blood cell count (WBC), even in the absence of diarrhea, *C. difficile* infection should be suspected.

#### Pseudomembranous colitis

**Fig. 3:**
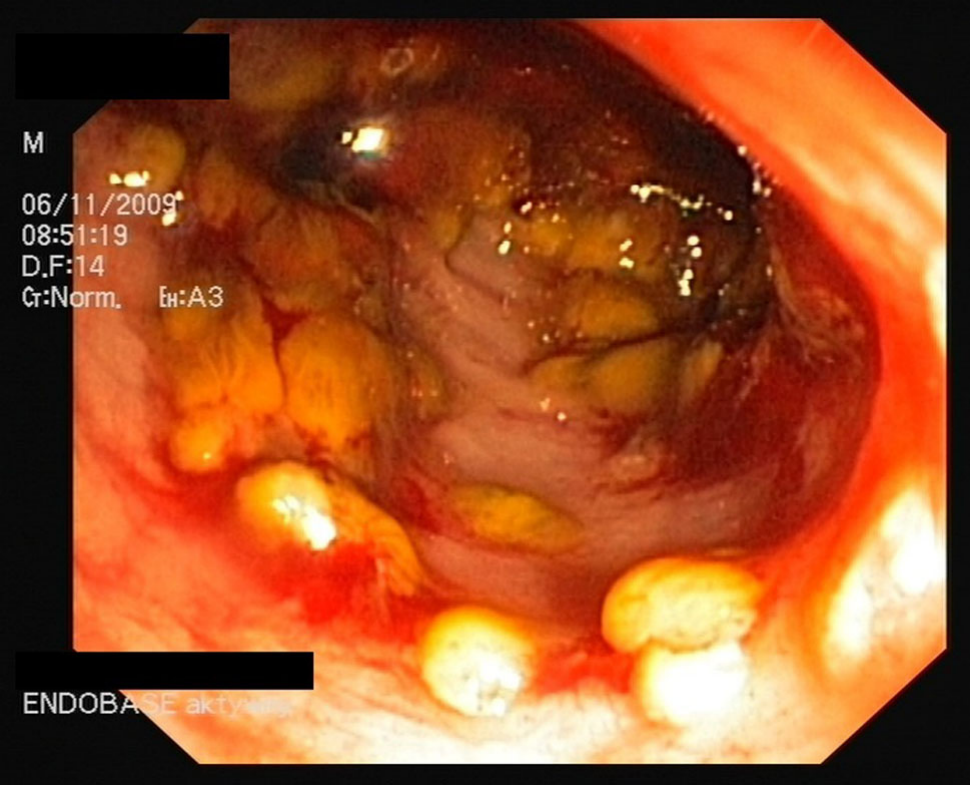
*C. difficile* pseudomembranous colitis

**Fig. 4:**
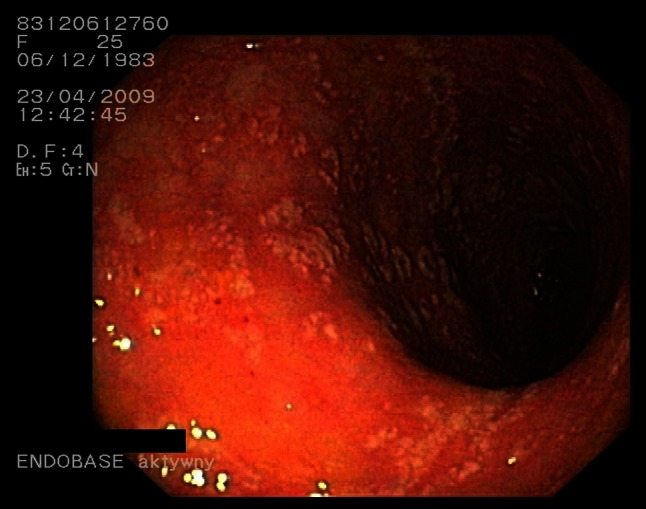
*C. difficile* pseudomembranous colitis

This is the most well-known form of *C. difficile* infection. During endoscopy, a characteristic yellow plaque can be observed in the mucosa of the colon and sometimes in the terminal ileum, which forms the basis for early diagnosis (Fig. 3). These plaques are small ulcerations of the mucous membranes, which trigger the release of serum proteins, mucus and inflammatory cells [6] (Fig. 4). Lymphocytes are found in biopsies of the lesion(s), and the patient may be classified into one of the three groups depending on the severity of infection (Table 2). Accompanying symptoms include severe abdominal pain, dehydration and often hypoalbuminemia (<30 mg/L). It is essential to initiate the appropriate medical treatment for patients with pseudomembranous colitis due to the potential toxic effects of the infection.

**Table 2:** Histopathologic severity of pseudomembranous colitis

Classification	Description of changes
Type 1	Mildest form, most of the changes are limited only to the superficial epithelium. Pseudomembranous changes are present, but ulcers are found only occasionally
Type 2	More evident changes in the colonic mucosa, gland disorders and significantly increased amount of secreted mucus. Inflammation that invades the basement membrane
Type 3	Full thickness necrosis is noticed within the whole bowel wall with pseudomembranous changes

Relapses occur in about 10–25 % of cured patients. Frequently, re-infection can be much more severe, and there is a greater predisposition to subsequent episodes of pseudomembranous colitis [19].

#### Fulminant colitis

**Fig. 5:**
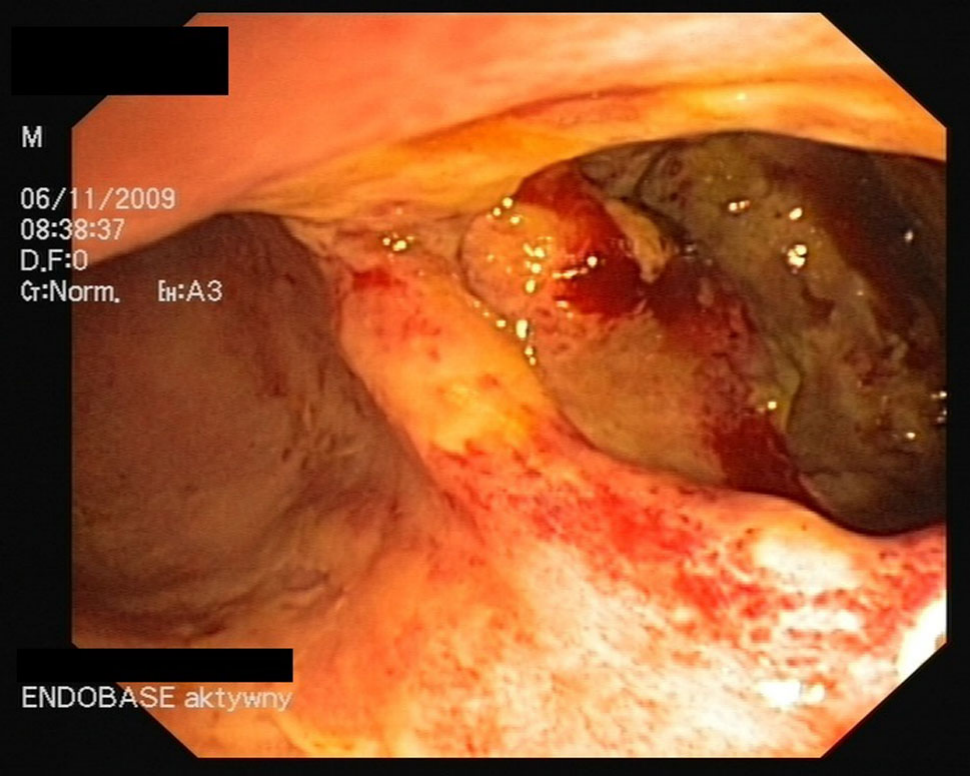
Perforation in *C. difficile* colitis

Generally, the natural progression of colitis allows one to conceive and follow a treatment plan; however, sometimes a fulminating form can develop. This form of inflammatory bowel disease develops only in 3–8 % of the patients [17]. A significant rise has been noted in recent years and is associated with a hypervirulent strain of the bacteria. This strain leads to the development of more systemic symptoms, multiple organ failure and overall increased mortality. Findings during physical examination include involuntary abdominal guarding. Full blood count analysis tends to show a marked leukocytosis (40 × 10^9^/L or more) [18] and anemia secondary to bleeding from gastrointestinal tract ulcers. Diarrhea can vary significantly, depending on the course of the disease, from a few episodes per day to complete obstruction and dilatation of the gastrointestinal tract. In the latter case, emergency surgical intervention is required as the mortality rate in these patients is very high and can reach 60 % [20], especially in older patients. If an inpatient has no history of *C. difficile* infection, diagnostic tests of the colon are still necessary. Stool cultures should be obtained. Colonoscopy should be performed by an experienced endoscopist in order to minimize the volume of air blown into the colon. Due to the risk of perforation, many doctors are wary of performing a colonoscopy during an acute episode of colitis. In fact, complications are quite rare (Fig. 5). The purpose of performing an urgent colonoscopy is not to evaluate the entire colon, but only to visualize the lining of the rectum and the distal colon. If obvious features indicative of fulminant colitis are found during the procedure, the endoscopist can simply remove the scope without having to examine the entire bowel [21].

#### *C. difficile*-associated enteritis

Inflammation of the small intestine in the course of *C. difficile* infection is quite rare. However, when it does occur, it tends to be seen in patients postcolectomy and in those with end ileostomies where high outputs are observed. In elderly patients who have multiple comorbidities, treatment in special referral centers is advisable [22].

#### Appendicitis

Appendicitis in the course of *C. difficile* infection is very rare. Up until 2007, only three cases have been described in the literature. However, the authors suggest that this complication may be significantly underdiagnosed, as many of the cases may have responded well to the conservative treatment, and no histology samples/specimens were available [23].

### Diagnosis

Diagnostic evaluation of stool samples should be carried out in patients with *C. difficile* infection who suffer from clinically significant diarrhea, i.e., 3 or more episodes of loose stool per day for 2 or more days. Other signs associated with diarrhea such as pyrexia and leukocytosis are suggestive of the diagnosis.

#### Laboratory tests

The easiest test is to detect the presence of toxins A and B in the stool sample. An enzyme immunoassay (EIA) has specificity up to 95 %, and the result is available after 4 h. However, the sensitivity is significantly reduced to 70–80 % due to the large number of false negatives [24]. The most reliable test is a stool culture. The sensitivity of this test reaches 90 %, but the results are not available for approximately 4–5 days. In addition to this time delay, not all laboratories routinely perform this assay. There are some molecular techniques available to identify the presence of the genome and its replication. These include polymerase chain reaction (PCR) ribotyping (a popular method because of its availability, efficiency and high specificity up to 97 % and sensitivity close to 91 %), pulsed-field gel electrophoresis (PFGE), multilocus variable number tandem repeat analysis (MLVA) and finally multilocus sequence typing (MLST) [25].

#### Endoscopy

Colonoscopy and sigmoidoscopy are both valuable diagnostic tools in diagnosing *C. difficile*. In the case of pseudomembranous colitis, the visible findings typically include a characteristic yellow plaque in the intestinal mucosa with ulceration and some associated bleeding. Table 3 shows the 4 main indications for colonoscopy according to Hookman’a et al. [26]. In a patient with the classical clinical symptoms and a positive stool culture, the endoscopic examination may be waived. In the case of fulminant colitis, it should, however, be performed, taking special care not to cause perforation.

**Table 3:** Indications for colonoscopy in the diagnosis of *C. difficile* infection

Indications for diagnostic colonoscopy
1. The results of the laboratory tests are negative, but there is a high probability of infection due to clinical symptoms
2. Earlier diagnosis required before the results of laboratory tests
3. Failure to respond to treatment with antibiotics
4. Atypical disease with obstruction and mild diarrhea

#### Imaging studies

A computed tomography (CT) scan of the abdomen and pelvis may be helpful in the advanced stages, when the wall of the intestine is characteristically thickened. Images are not specific for bacterial infections [27].

### Treatment

*Clostridium difficile* infection is one of the most common causes of nosocomial infections and in particular is responsible for increased morbidity and mortality in elderly patients. The bacteria colonize the gastrointestinal tract, when the physiological bacterial flora is disrupted due to the use of antibiotics. Treatment of the infection depends on the severity of the disease and the symptoms. It can be treated conservatively or with surgery.

The most important initial treatment step is to cease administration of the antibiotic that caused the *C. difficile* infection (CDI). The continuous administrations of antibiotics, which do not treat *C. difficile*, not only worsen the patient’s condition, but may also affect their susceptibility to re-infection [28]. If, due to the primary disease, administration of antibiotics is required, it would be prudent to incorporate antibiotics that are less responsible for extending CDI, such as aminoglycosides, sulfonamides, macrolides, tetracyclines and vancomycin. The general principles of prevention of further infections should also be kept in mind. Patients with suspected or confirmed CDI should be placed in isolation because of the high risk of contamination. All medical staff should perform thorough hand hygiene following contact with patients and also sanitize any medical equipment used, e.g., stethoscope. Patients should be well hydrated, and their electrolyte levels should be carefully monitored. There are no dietary limitations with the exception of patients who have surgery already scheduled. In patients with typical symptoms of CDI, such as diarrhea, abdominal pain, nausea and positive stool cultures, antibiotics should be initiated [29]. Empirical therapy is only indicated when there is a very high probability of infection and while awaiting the results of diagnostic tests. Carriers should not be treated with antibiotics when there are no clinical symptoms of the infection.

In the treatment of less severe infections, initial therapy should consist of metronidazole or vancomycin. Several randomized clinical trials showed significant efficacy of these antibiotics in the treatment of CDI [30]. Both drugs in a study by Zar and colleagues [30] showed similar efficacy (90–98 %), but metronidazole is considered to be the drug of choice. The advantages of metronidazole are much lower costs of the therapy and reduced spread of vancomycin-resistant enterococci (VRE). Metronidazole should be prescribed at a dose of 500 mg 3 times daily or 250 mg 4 times a day for 10–14 days. The oral dose of vancomycin is 125 mg every 6 h. The use of higher doses such as 500 mg has no documented association with a shorter recovery time. Intravenous forms of the medication are not used as they have poor penetration into the gastrointestinal tract [31]. Further examination of stool samples during treatment is not indicative of response to treatment as approximately 50 % of patients may have positive results up to 6 weeks after cessation of treatment [31]. The use of monoclonal antibodies against toxins A and B is a subject of a great interest and hope. Currently they are not used or recommended for routine treatment. Lowy et al. [32] studied 200 patients to whom standard doses of antibiotics were given (101 in the antibody group and 99 in the placebo group). The incidence of relapse was 7–25 %. More randomized trials are needed to determine the benefit of this therapy as a standard procedure.

Patients with severe infections may develop systemic failure with copious diarrhea and must be treated in the intensive care unit or surgical ward. There is no general classification of “severity of infection” available, but in the literature, a number of signs and symptoms are reported that indicate which patients are candidates for intensive therapy. These include white count C >15 × 10^9^/L, elevated creatinine, temperature >38.8 °C and albumin <2.5 mg/dL. Generally, the physicians clinical decision and opinion are considered to be the most important when initiating intensive treatments. Recent guidelines from 2010 [33] recommend vancomycin as the drug of choice. Its main advantage is that vancomycin given orally is not absorbed by the body so the full dose reaches the large intestine, the site of infection. Many clinicians use Vancomycin per os at a dose of 500 mg 4 times daily despite a lack of evidence for the effectiveness of this therapy. The recommended dose is still 125 mg 4 times daily [33]. In patients refractory to this treatment, fidaxomicin or metronidazole can be used. In patients with a bowel obstruction, vancomycin enemas with continuous oral [34] or intravenous therapy, plus metronidazole every 8 h is recommended.

Patients with severe and protracted infections can sometimes develop rectal toxicity (toxicum megacolon), perforation and necrosis of the intestine or rapidly progressive infections with multiple organ failure. These patients require surgical intervention [35]. The optimal time for surgery is within 48 h of initiating conservative treatment without seeing a response, the development of multiple organ failure or a bowel perforation [20]. The difficulty is in determining the optimal time for surgical intervention as not all patients will survive the initial 48 h. Considerations also need to be made regarding cases of bowel obstruction and persistent diarrhea and vomiting which are not suitable for conservative treatment. The Canadian retrospective study already referred to [21] showed that a colectomy was the most beneficial treatment in patients above 65 years of age, with a WBC >20 × 10^9^/L and elevated serum lactate between 2.2 and 4.9 mmol/L. Positive peritoneal signs, obstruction, perforation and signs of toxic megacolon should also be included in these criteria.

CDI infections are currently treated by two different surgical approaches. One is subtotal colectomy. This involves the removal of the entire colon with the creation of an ileostomy, leaving the rectum in place. The other procedure is less invasive—a diverting loop ileostomy with colonic lavage.

#### Subtotal colectomy

The number of subtotal colectomies performed continues to escalate due to the presence of hypervirulent strains of *C. difficile*. Currently, about 5 % of patients infected with *C. difficile* reach the stage of fulminant colitis and undergo surgery. The procedure selected and the outcome depends on the level of experience of the surgeon, but better results are obtained in the case of total colectomy [36]. Earlier surgical intervention is also associated with better results. An emergency colectomy for advanced forms of *C. difficile* is associated with higher mortality rates. Al-Abed et al. [37] operated on 3.7 % of his patients with an associated mortality rate of over 40 %. The majority of patients who had significant comorbidities (75 %) did not survive after an emergency colectomy. Anton D. Parera et al. [38] had very similar results. The 30-day mortality rate was 45.7 %. Therefore, it is crucial to identify infected patients early before they progress into fulminant colitis and organ failure.

#### Diverting loop ileostomy with colonic lavage

This procedure may be an interesting alternative to a colectomy. Neal et al. [39] operated on 42 patients using this procedure. Comparing colectomy and diverting loop ileostomy with lavage, the authors noted a reduction in mortality from 50 to 19 %. After creating the ileostomy, the colon may be flushed with warm polyethylene glycol. Postoperatively vancomycin enemas can be administered via the ileostomy. This procedure was performed laparoscopically in 32 patients, which was equally effective and less harmful to the patient at the same time. This is especially important among elderly patients.

#### Fecal transplant

As previously mentioned, a disruption in the balance of the normal intestinal flora is a major risk factor for *C. difficile* infection and indeed recurrent infections. In several uncontrolled trials, the administration of stool from a healthy donor has been used with a high degree of success [40]. If a “fecal transplant” is considered, the donor should be screened for transmissible diseases. Logistic issues to be considered include timing, collection and processing of the specimen from the donor. The feces can be delivered via nasogastric tube or enema.

#### New treatments

Some new, interesting therapies are being tried and tested in *C. difficile* treatment. Vancomycin therapy followed by rifaximin may be effective in the treatment of *C. difficile* infection. In one series, 8 patients received a 2-week course of rifaximin once they were clinically asymptomatic, after the last administration of vancomycin. Seven patients had no further recurrence of infection [41]. Also, intravenous immunoglobulin (IVIG) containing *C. difficile* antitoxin has been used in some patients with severe *C. difficile* colitis. A retrospective review with a comparison to some case reports revealed that there is no significant difference in the clinical outcomes [42]. Probiotics may also be effective in the prevention and treatment of CDAD, in several ways: the alteration of intestinal flora, increased antimicrobial activity, intestinal barrier protection and immunomodulation. The clinical role of this therapy is an evolving area of study [43].

## Discussion

In recent years, the number of nosocomial infections has risen significantly. This is almost certainly because of the overprescribing of antibiotics and one could certainly question the clinical indications for said use. Also to blame are the lengthy admission stays in surgical departments and intensive care units. The demographics of the surgical patient have changed in recent times, and there are now more elderly and medically complex patients. Several years ago, this subset of the population may have been deemed unsuitable for surgical intervention and therefore exempt from procedures. As we now routinely treat an increasingly elderly subset of patients, we have to allow for the fact that their average length of hospital stay is longer than that of other patients. This group would realistically stay more than 7 days as inpatients, thereby running a major risk of contracting a *C. difficile* infection. In these cases, preventative measures, a heightened level of awareness and knowledge of early clinical symptoms are of vital importance.

Obviously with the ever increasing severity of the infection comes an associated increase in morbidity and mortality. There are numbers of independent predictors of mortality for CDAI (Table 4). Patients, who present with these risk factors, especially with strong predictors, should have early surgical consultations and early aggressive surgical intervention should be considered.

**Table 4:** Predictors of mortality for fulminant *C. difficile* colitis

Strong predictors of mortality for fulminant *C. difficile* colitis
1. Age ≥70 years
2. Severe infection WBC ≥35,000 or ≤4,000/μL or neutrophil bands ≥10 %
3. Need for cardiorespiratory support (vasopressin or intubation)
4. Arterial lactate >4.9
5. Mental status change
Weak predictors of mortality for fulminant *C. difficile* colitis
1. Type of surgery (total colectomy vs. segmental resection)
2. Delayed surgical intervention
3. Admission to other than surgical ward
4. Multiple comorbidities
5. No vancomycin use during medical treatment

*WBC* white cell count

The choice of initial treatment, surgical versus medical, and the type of surgical resection influence the final outcomes (Table 5).

**Table 5:** Morbidity related to *C. difficile* infection and treatment

Morbidity after fulminant *C. difficile* colitis (%)	
Overall 30-day mortality	34–57
5-Year survival rates	16.3–38
Subtotal colectomy and end ileostomy mortality rate	11
Segmental colectomy mortality rate	42–100
Diverting loop ileostomy with colonic lavage mortality rate	19
Stoma reversal rate	20

**Fig. 6:**
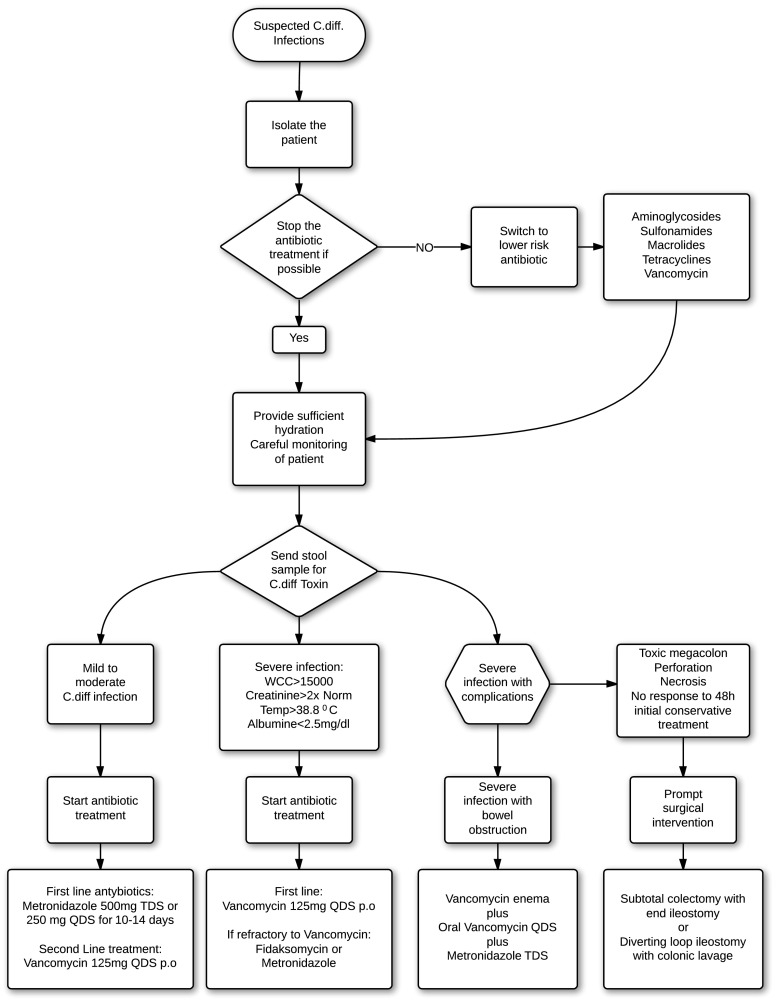
Clinical approach to treatment of *C. difficile* infection

If we take into account the lack of specific guidelines for the treatment of *C. difficile* infection, and the number of currently available surgical techniques, physicians and surgeons have a considerable range of potential approaches to use. Figure 6 shows the clinical approach to treatment of *C. difficile* infection.

## Conclusions

Despite the overall positive results following a subtotal colectomy, recent research shows that there is potential to develop safer and less invasive techniques. The authors of this study would like to highlight the ever increasing and problematic issue of rising levels of nosocomial infections. Further research is of paramount importance to help reduce their occurrence.
